# Novel Poly(Methylenelactide-*g*-L-Lactide) Graft Copolymers Synthesized by a Combination of Vinyl Addition and Ring-Opening Polymerizations

**DOI:** 10.3390/polym13193374

**Published:** 2021-09-30

**Authors:** Tanyaluck Mekpothi, Puttinan Meepowpan, Montira Sriyai, Robert Molloy, Winita Punyodom

**Affiliations:** 1 Department of Chemistry, Faculty of Science, Chiang Mai University, Chiang Mai 50200, Thailand; tanyaluck01@hotmail.com (T.M.); pmeepowpan@gmail.com (P.M.); 2 Center of Excellence for Innovation in Chemistry (PERCH-CIC), Faculty of Science, Chiang Mai University, Chiang Mai 50200, Thailand; 3 Bioplastics Production Laboratory for Medical Applications, Faculty of Science, Chiang Mai University, Chiang Mai 50200, Thailand; msriyai@yahoo.com; 4 Center of Excellence in Materials Science and Technology, Chiang Mai University, Chiang Mai 50200, Thailand; bob.chiangmai@gmail.com; 5 Materials Science Research Center, Faculty of Science, Chiang Mai University, Chiang Mai 50200, Thailand

**Keywords:** vinyl addition polymerization, ring-opening polymerization (ROP), bromolactide, methylenelactide, poly(methylenelactide), poly(methylenelactide-*g*-L-lactide), thermal stability

## Abstract

In this work, a novel poly (methylenelactide-*g*-L-lactide), P(MLA-*g*-LLA) graft copolymer was synthesized from poly(methylenelactide) (PMLA) and L-lactide (LLA) using 0.03 mol% liquid tin(II) *n*-butoxide (Sn(O*n*Bu)_2_) as an initiator by a combination of vinyl addition and ring-opening polymerization (ROP) at 120 °C for 72 h. Proton and carbon-13 nuclear magnetic resonance spectroscopy (^1^H- and ^13^C-NMR) and Fourier-transform infrared spectroscopy (FT-IR) confirmed the grafted structure of P(MLA-*g*-LLA). The P(MLA-*g*-LLA) melting temperatures (*T*_m_) range of 144–164 °C, which was lower than that of PLA (170–180 °C), while the thermal decomposition temperature (*T*_d_) of around 314–335 °C was higher than that of PLA (approx. 300 °C). These results indicated that the grafting reaction could widen the melt processing range of PLA and in doing so increase PLA’s thermal stability during melt processing. The graft copolymers were obtained with weight-average molecular weights (${\bar{M}}_{w}$) = 4200–11,000 g mol^−1^ and a narrow dispersity (*Đ* = 1.1–1.4).

## 1. Introduction

Poly(lactic acid) or polylactide (PLA), an aliphatic polyester derived from renewable starch-containing resources such as corn; sugar beet; and cassava, is one of the most significant biodegradable polymers extensively used as a petroleum-based materials replacement due to its biodegradability and environmentally-friendly properties [1,2,3,4,5,6,7,8,9,10,11]. However, it has been commonly known that PLA shows brittleness with poor melt strength and thermal stability which limit its processability and performance in desired applications [12,13,14,15,16,17,18,19,20]. For instance, PLA has been reported to show a rather low heat deflection temperature (HDT) of approximately 55 °C because of the low degree of crystallinity of PLA materials resulting from the low crystallization rate while processing [21,22,23,24,25]. Since these drawbacks in toughness and heat resistance confine specific applications of PLA, thereby, many attempts for improving PLA inferior properties via various modification methods have been investigated over the past decades [26,27]. The strategies include annealing, nucleating agents, plasticization, chain extending, copolymerization, blending, polymer (nano)composites, stereocomplexes, crosslinking, and functionalization [27,28,29,30,31,32,33].

For the modification of PLA by copolymerization, most studies have been paying attention to the copolymerizing of the lactide with either lactide/lactone monomers or other monomers with functional groups such as amino and carboxylic groups, etc [34,35,36]. The copolymers such as poly(lactide-*co*-glycolide) (PLGA), poly(lactide-*co*-*ε*-caprolactone) (PLCL), poly(lactic acid)-poly(ethylene oxide) (PLA-PEO) and poly(lactic acid-glutamic acid) (PLGM) are generally synthesized via ring-opening polymerization (ROP) of cyclic ester monomers [37,38,39]. These copolymers could synergistically combine the advantages of both components showing greater properties, thus, overcome the limitations of the individual ones. Similar to the homopolymerization process, the copolymerization consists of three stages: (1) initiation, (2) propagation, and (3) termination [40]. The copolymer molecules contain two different repeating units in which different monomers can be arranged in various types such as random, alternating, block and graft copolymers [41]. As such, a graft copolymer is one of the most widely used methods for a modification of PLA to achieve improved properties such as processability and physicochemical properties since it can produce a large variety of polymers with different properties for widely used in many applications [42,43,44,45]. For the synthesis of a well-defined graft copolymer, there are substantial difficulties in achieving precise control over its specific end groups, microstructure, and composition [46]. Therefore, the experimental conditions must be optimized in order to obtain the suitable polymerization process for the designed system.

*O*-heterocyclic monomers such as lactide (LA) and methylenelactide (MLA) functionalities are of interest to be used as functional materials due to their potential to be selectively polymerized via either vinyl-addition or ring-opening pathways [47,48,49]. Since PLA does not bear any functional groups, many efforts for synthesizing new alkyl modified lactides have been made aiming to modulate the physicochemical properties such as glass transition temperature (*T*_g_), toughness, thermal stability, and processability [34,50,51,52,53]. However, certain endeavors for chemical modifications of lactide are scarce because of fast ester bonds hydrolysis [54,55,56].

In contrast to lactide, methylenelactide (MLA, 3-methylene-6-methyl-1,4-dioxane-2,5-dione) represents a free radical polymerizable derivative of lactide. In 1969, the synthesis of a methylene-functional lactide from the monobromination of lactide using *N*-bromosuccinimide (NBS) followed by base-catalysed dehydrobromination (HBr elimination) was studied for the first time by Scheibelhoffer et al. [57]. Moreover, from the work of Miyake and coworkers [48], it was found that MLA could be polymerized by a radical vinyl-addition. ROP pathways, however, resulted in monomer decomposition to produce nonpolymerizable derivatives instead. Nonetheless, Jiang and Hillmyer [54] showed that further derivatization of MLA could provide functional monomers for ROP using MLA as a dienophile in a Diels–Alder reaction with cyclopentadiene. The resultant tricyclic product could be polymerized by either ROP of the lactide ring or via ring-opening metathesis polymerization (ROMP). Afterwards, Hillmyer and coworkers continued exploring other dienes to gather more data on functional lactide monomers, and further polymerization provided new polyesters with diverse thermal properties [55].

Recently, Britner and Ritter [58,59] reinvestigated Scheibelhoffer’s first study of a radical polymerizable lactide derivative with respect to polymerization and polymer analogous aminolysis under mild conditions. In this work, the optically active MLA was polymerized by free radical polymerization in solution using AIBN as an initiator and resulted in isotactic-biased atactic optically active poly(methylenelactide) (PMLA) with molecular weights ranging between 4 × 10^4^–1 × 10^5^ g mol^−1^ with dispersity, *Đ*~2.5 and a *T*_g_ of 244 °C. Britner and Ritter also performed a further study on free-radical polymerization, copolymerization, and controlled radical polymerization of the cyclic monomer MLA in comparison to the non-cyclic monomer *α*-acetoxyacrylate (AA). The results showed that MLA undergoes a self-initiated polymerization. Moreover, in this study, reversible addition fragmentation chain transfer (RAFT)-controlled homopolymerization of MLA and copolymerization with *N*,*N*-dimethylacrylamide (DMAA) was performed at 70 °C in 1,4-dioxane using AIBN and 2-(((ethylthio)carbonothioyl)thio)-2-methylpropanoic acid as an initiator and a transfer agent respectively.

Derivatization of MLA via the Diels–Alder cycloaddition with dienes leads to bifunctional monomers that can be polymerized into various functionalized PLA materials. The need for functionalized PLA materials with enhanced physical and mechanical properties has led to an effective strategy to derivatize PLA, which is synthesized through a bromination of L-lactide (LLA) with *N*-bromosuccinimide using benzoyl peroxide as an initiator and followed by a basic HBr elimination with Et_3_N in dichloromethane to produce a monomer MLA. After that, MLA was used as a substrate of PMLA synthesis which was then synthesized through a vinyl-addition polymerization with AIBN initiator via the free radical polymerization. According to previous works, PMLA has poor solubility, sluggish dissolution, and that it is challenging to handle the polymer as a melt for polymerization. As previously mentioned, Britner et al. [58,59] have found a way to overcome this challenge by PMLA functionalization. In the underlying study, Britner and Ritter focused on spatial effects with respect to interactions between neighboring lactide rings. Moreover, it was found that the PMLA attached lactide rings react like activated esters and thus readily undergo quantitative amidation reactions with aliphatic primary amines under mild conditions. However, up to present, no information on the copolymerization of MLA and LLA via the ROP has been reported [54,55,56,58,59,60,61,62].

**Scheme 1:**
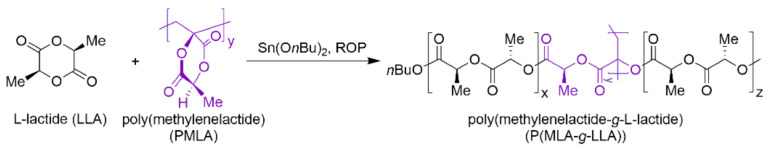
The synthesis of poly(methylenelactide-*g*-l-lactide), P(MLA-*g*-LLA), via ROP.

Accordingly, the main objective of this study was to synthesize poly(methylenelactide-*g*-L-lactide), P(MLA-*g*-LLA), graft copolymers from L-lactide (LLA) and PMLA via ROP in solution using a novel soluble liquid tin(II) *n*-butoxide (Sn(O*n*Bu)_2_) as an initiator [63,64,65,66,67,68]. These materials have not been previously reported in the literature. The synthesis reaction of a P(MLA-*g*-LLA) via ROP and its chemical structure are shown in Scheme 1. The intended application of these graft copolymers is as potentially reactive, network-bound (by transesterification) additives to improve the melt processability and thermal properties of commercial PLA.

## 2. Materials and Methods

### 2.1. Materials

L-lactide (LLA) and liquid tin(II) *n*-butoxide (Sn(O*n*Bu)_2_) were purchased from the Bioplastics Production Laboratory for Medical Applications (ISO 13485:2016 TÜV SÜD America Accredited Laboratory, Chiang Mai University, Chiang Mai, Thailand) and used as received [64]. Benzoyl peroxide (75%), triethylamine (≥99%), calcium hydride (90–95%), and azobisisobutyronitrile (AIBN) were purchased from Sigma-Aldrich (Saint Louis, MO, USA). *N*-bromosuccinimide (99%) was purchased from Acros (Geel, Belgium). Magnesium sulfate anhydrous (96%) was purchased from PanReac AppliChem (Barcelona, Spain). Sodium bisulfite (58.5–67.4%) was purchased from HiMedia (Mumbai, India). 1,2-Dichlorobenzene (≥98%) was purchased from Carlo Erba (Milano, Italy). Chloroform-*d*_1_ (99.8% CDCl_3_) and dimethyl sulfoxide-*d*_6_ (99.9% DMSO) were purchased from CIL (Kolkata, India). Chloroform (99.8%) and hydrochloric acid (37%) were purchased from RCI Labscan (Bangkok, Thailand). Methanol, dichloromethane, ethyl acetate, and *n*-hexane (RCI Labscan) were purified by distillation prior to use.

### 2.2. Methods

In this study, bromolactide (Br-LA) and methylenelactide (MLA) were synthesized according to literature procedures reported by Britner and Ritter [58] with minor modifications.

#### 2.2.1. Synthesis of Bromolactide (Br-LA)

l-lactide (120.0 g, 832.6 mmol), *N*-bromosuccinimide (164.0 g, 921.4 mmol), and dichloromethane (600 mL) were added into a 1 L three-neck round-bottom flask equipped with a magnetic stirring bar, septum stoppers, and a reflux condenser. The reaction mixture was refluxed at 80 °C with continuous stirring, benzoyl peroxide (4.020 g, 16.60 mmol) in a 60 mL dichloromethane was then gradually added to the mixture solution. The mixture continued to be heated under reflux at 80 °C for 72 h and allowed to cool to room temperature. The precipitate was filtered off through the filter paper several times until a clear filtrate was obtained. The filtrate was then vacuum dried using a rotary evaporator (Heidolph, Hei-VAP Core, Schwabach, Germany) to yield a yellow solid residue. The yellow solid was dissolved in a 450 mL dichloromethane before pouring into a separatory funnel and washing with 0.2 M sodium bisulfite three times (3 × 1000 mL) and once (900 mL) with saturated sodium chloride. Subsequently, the organic layer was dried over MgSO_4_ and evaporated under reduced pressure to obtain a pale yellow needle-like crystal. The Br-LA product was then purified by recrystallization from the mixture of dichloromethane and *n*-hexane (1:1), filtered off, and vacuum dried at room temperature.

#### 2.2.2. Synthesis of Methylenelactide (MLA)

For the preparation of methylenelactide (MLA), 10.00 g (44.84 mmol) of the synthesized Br-LA product from Section 2.2.1 and 20 mL of dichloromethane were added into a 250 mL three-neck round-bottom flask. The solution was stirred under an inert (N_2_) atmosphere and cooled to 0 °C using an ice bath. Triethylamine (2.0 mL, 14.33 mmol) was then added dropwise to a solution and the mixture was kept stirring at 0 °C for 2 h and at room temperature for 1 h. The reaction mixture was washed with 150 mL of 1 M HCl (three times) and saturated sodium chloride (once) successively, dried over MgSO_4_, evaporated, and dried under vacuum to obtain the MLA product as a yellowish-brown solid.

#### 2.2.3. Synthesis of Poly(methylenelactide) (PMLA)

In this work, the preparation of poly(methylenelactide) (PMLA) was followed the method as described by Miyake et al. [48]. Firstly, a 100 mL round-bottom flask equipped with a magnetic stirring, methylenelactide (4.000 g, 28.15 mmol) and 50 mL of 1,2-dichlorobenzene (*o*-DCB) was added and the reaction mixture was purged with N_2_ for 30 min. Then, various required amounts of AIBN (5, 10, 15, 20, and 25 mol%) was added and the flask was sealed with a rubber septum. The reaction flask was then immersed in a silicone oil bath at 75 °C for 1 h. The reaction solution was quench cooled and then poured into 800 mL of methanol to obtain the polymer precipitate. The PMLA polymer was isolated by filtration, washed extensively with methanol and dried to constant weight in a vacuum oven at 50 °C overnight to constant weight.

#### 2.2.4. Synthesis of Poly(methylenelactide-*g*-l-lactide) (P(MLA-*g*-LLA)) via Solution Polymerization

Poly(methylenelactide-*g*-l-lactide) (P(MLA-*g*-LLA) with the composition ratio of PMLA to LLA = 1:30 and 1:50 *w*/*w* and the composition ratio of 1:300, 1:600 *w*/*w* for PMLA (20 mol% AIBN) was synthesized via ring-opening polymerization (ROP). Firstly, PMLA and LLA with 0.03 mol% liquid tin(II) *n*-butoxide (Sn(O*n*Bu)_2_) initiator, 2.0 mL of 1,2-dichlorobenzene (*o*-DCB), and a magnetic stirring bar were loaded into a round-bottom flask. The solution polymerization of P(MLA-*g*-LLA) was carried out at 120 °C for 72 h, after which, the graft copolymers were purified by methanol. The graft polymer was again isolated by filtration, washed with more methanol, and dried in a vacuum oven at 50 °C overnight to constant weight.

#### 2.2.5. Synthesis of Poly(l-lactide) (PLA) via Solution Polymerization

l-lactide (10.00 g) with 0.03 mol% of liquid Sn(O*n*Bu)_2_ initiator, 5.0 mL of 1,2-dichlorobenzene (*o*-DCB), and a magnetic stirring bar were loaded into a round-bottom flask. Solution polymerization of PLA was carried out at 120 °C for 72 h under vacuum. The crude PLA product was purified by precipitation in methanol, in filtration, washing with more methanol, and drying in a vacuum oven at 50 °C overnight to constant weight.

### 2.3. Instrumental Methods

IR spectra were recorded using an attenuated total reflectance (ATR-FTIR model Nicolet^TM^ iS5 (Thermo Scientific^TM^, Waltham, MA, USA) spectrometer and the wavenumbers of maximum absorption peaks are reported in cm^−1^. ^1^H-NMR (400 MHz) and ^13^C-NMR (100 MHz) were recorded on a Bruker DRX-400 spectrometer (Billerica, MA, USA). Where necessary, 2D NMR experiments (HMBC and HBQC) were carried out to assist the structure elucidation and signal assignments. Chemical shifts (*δ*) were reported in ppm down-field from TMS as an internal reference or with the solvent resonance as the internal standard (CHCl_3_ impurity in CHCl_3_-*d*_1_, δ 7.26 and 77.0 ppm; DMSO impurity in DMSO-*d*_6_, 2.50 and 39.52 ppm); data are reported in the following order: chemical shift, multiplicity and coupling constants (*J*) are given in hertz. Splitting patterns are indicated as *s*, singlet; *d*, doublet; *t*, triplet; *m*, multiplet; *q*, quartet for ^1^H-NMR data. Broad peaks are denoted by br before the chemical shift multiplicity. Gel permeation chromatograph (GPC) analyses were carried out on Waters 2414 refractive index (RI) detector, equipped with Styragel HR5E 7.8 × 300 mm column (molecular weight resolving range = 2000–4,000,000). The GPC columns were eluted using tetrahydrofuran (THF) with a flow rate of 1.0 mL min^−1^ at 40 °C with an injection volume of 100 µL, a 30 min run time, and a toluene flow marker. Number-average molecular weights (${\bar{M}}_{n}$) and dispersity (*Đ*) of the polymers were determined based on calibration with polystyrene (PS) standards using Empower 3 Software. DSC measurements were performed on a Mettler-Toledo DSC-1 Differential Scanning Calorimeter (Columbus, OH, USA) under a flowing nitrogen (N_2_) atmosphere (20 mL min^−1^). Prior to measurement, the instrument was calibrated using high purity (>99.999%) indium and tin standards and the data obtained were analyzed by using STARe Software v12.10. For each experiment, 3–5 mg of the polymer sample was weighed into a 50 mL aluminium pan and hermetically sealed. Measurements were performed at a heating rate of 10 °C min^−1^ over the temperature range of 0 to 280 °C for two heating cycles. In the first scan, the polymer was heated from 0 to 280 °C at a heating rate of 10 °C min^−1^, hold at 280 °C for 3 min, then cooled to 0 °C at 10 °C min^−1^, hold at this temperature for another 3 min, and reheated to 280 °C at a ramp of 10 °C min^−1^. All of the transition temperature (i.e., *T*_g_, *T*_c_, and *T*_m_) values were obtained from the second scan after eliminating thermal history in the first scan. Degradation temperature (*T*_d_) values of the polymers were measured by thermal gravimetric analysis (TGA) method on a Perkin-Elmer TGA7 Thermogravimetric Analyzer (Pyris 1 Software). The TGA temperature and weight calibrations were performed using magnetic transition temperature standards: alumel, nickel, and perkalloy (*T*_onset_ = 154.20, 355.30, and 596.00 °C) with high purity of 99.999% and a stainless steel standard weight (100 mg) respectively. For all measurements, the temperature range of 50 to 550 °C was scanned at a heating rate of 20 °C/min under dry N_2_ purge gas.

## 3. Results and Discussion

### 3.1. Synthesis of Bromolactide (Br-LA)

The purified Br-LA product of >99.5% purity (DSC) was obtained as a white, needle-like crystals in a percentage yield of ~60%. DSC results showed a narrow melting peak (*T*_m_) range of 104–112 °C. ^1^H-NMR (400 MHz, CDCl_3_, δ): 7.26 (q, *J* = 5.50 Hz, 1H; CH), (s, *J* = 2.33 Hz, 3H; CH_3_), (d, *J* = 1.73 Hz, 3H; CH_3_); ^13^C-NMR (100 MHz, CDCl_3_, δ): 164.95 (C=O), 162.13 (C=O), 81.52 (quaternary carbon C), 73.87 (CH), 29.82 (CH_3_), 16.60 (CH_3_).

### 3.2. Synthesis of Methylenelactide (MLA)

The synthesized MLA product was obtained as a pale yellow solid (60% yield). However, a rather broad melting temperature range of 77–132 °C was observed indicating the purity of < 95%. ^1^H-NMR (400 MHz, CDCl_3_, δ): 7.26 (d, *J* = 5.95 Hz, 2H; CH_2_), (d, *J* = 5.55 Hz, 2H; CH_2_), (q, *J* = 5.04 Hz, 1H; CH), (m, *J* = 1.71 Hz, 3H; CH_3_); ^13^C-NMR (100 MHz, CDCl_3_, δ): 190.99 (C=O), 175.25 (C=O), 159.83 (quaternary carbon C), 110.68 (CH_2_), 69.86 (CH), 16.71 (CH_3_).

### 3.3. Synthesis of Poly(methylenelactide) (PMLA)

**Scheme 2:**
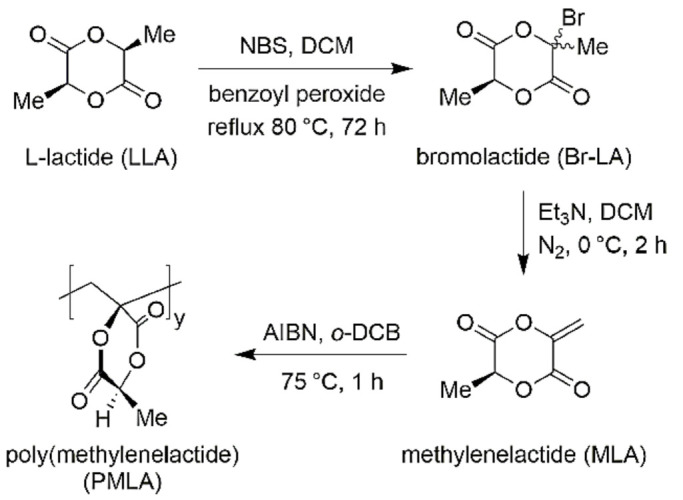
Synthetic pathway of poly(methylenelactide) (PMLA).

The synthetic pathway of PMLA is as shown in Scheme 2. From Scheme 2, it can be seen that, in the first step, Br-LA was synthesized via the bromination of LLA with *N*-bromosuccinimide using benzoyl peroxide as an initiator. The effect of the bromination reaction was the production of strong acids of hydrobromic acid (HBr) which was removed by using an excess of 0.2 M sodium bisulfite solution. Then, the MLA was synthesized via the elimination of Br-LA with triethylamine in dichloromethane. After that, the MLA was used as an initial substance in the synthesis of PMLA. It was found that PMLA was obtained as a white solid with a % yield of 60–65%. ^1^H-NMR (400 MHz, DMSO-*d*_6_, δ):2.50 (broad q, *J* = 5.45–5.46 Hz, 1H; CH), (broad s, *J* = 3.35–3.37 Hz, 2H; CH_2_), (broad d, *J* = 1.41–1.43 Hz, 3H; CH_3_); ^13^C-NMR (100 MHz, DMSO-*d*_6_, δ): 166.50 (C=O), 163.74 (C=O), 80.76 (CH), 72.77 (main-chain quaternary carbon C), 45.08 (CH_2_), 18.63 (CH_3_), which ^1^H- and ^13^C-NMR spectral data are identical to those previously published [48]; FT-IR (cm^−1^): 2449 and 1447 (w), 1747 (s), 1258 (s).

### 3.4. Synthesis of Poly(methylenelactide-g-l-lactide) (P(MLA-g-LLA))

**Scheme 3:**
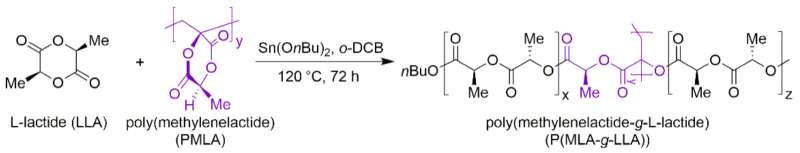
Synthesis of poly(methylenelactide-*g*-l-lactide) (P(MLA-*g*-LLA)).

Scheme 3 shows the synthetic pathway of P(MLA-*g*-LLA). The physical appearances of the purified P(MLA-*g*-LLA) products from solution polymerization with the composition ratios of PMLA:LLA of 1:30 and 1:50 *w*/*w* were as white solids in ~90% yield. The copolymers were characterized in terms of their chemical structure by ^1^H-NMR. ^1^H-NMR (400 MHz, CDCl_3_, δ): 7.26 (q, *J* = 5.15 Hz, 1H; CH), (q, *J* = 4.34 Hz, 1H; CH), (broad m, *J* = 2.75–4.25 Hz, 2H; CH_2_), (m, *J* = 1.57 Hz, 3H; CH_3_); ^13^C-NMR (100 MHz, CDCl_3_, δ): 174.94 (C=O), 173.36 (C=O), 169.41 (C=O), 69.01 (CH), 68.77 (CH_2_), 68.18 (main-chain quaternary carbon C), 66.71 (CH), 20.30 (CH_3_), 16.45 (CH_3_); FT-IR (cm^−1^): 2997 and 1455 (w), 1755 (s), 1087 (s).

### 3.5. Synthesis of Poly(L-Lactide) (PLA)

PLA was obtained as a white solid with a % yield of ~90%. ^1^H-NMR (CDCl_3_, 400 MHz): δ = 7.26 (q, *J* = 5.16 Hz, 1H; CH), (d, *J* = 1.58 Hz, 3H; CH_3_) ppm; ^13^C-NMR (CDCl_3_, 100 MHz δ): 169.73 (C=O), 69.14 (CH), 16.78 (CH_3_) ppm; FT-IR (cm^−1^): 2997 and 1455 (w; ν (C–H)), 1754 (s; ν (C=O)), 1085 (s; ν (C–O)).

### 3.6. Characterization of PMLA, PLA, and P(MLA-g-LLA)

**Figure 1:**
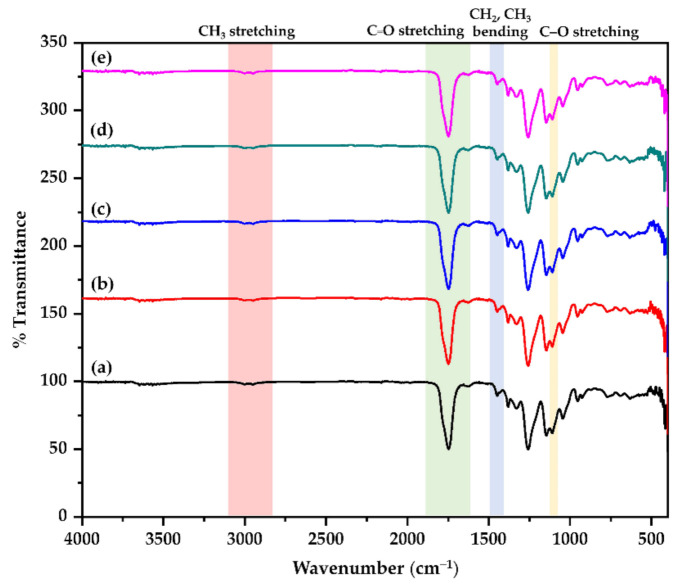
FT-IR spectra of poly(methylenelactide) with different amounts of AIBN (5, 10, 15, 20, and 25 mol%): (**a**) PMLA_5_; (**b**) PMLA_10_; (**c**) PMLA_15_; (**d**) PMLA_20_; and (**e**) PMLA_25_.

**Figure 2:**
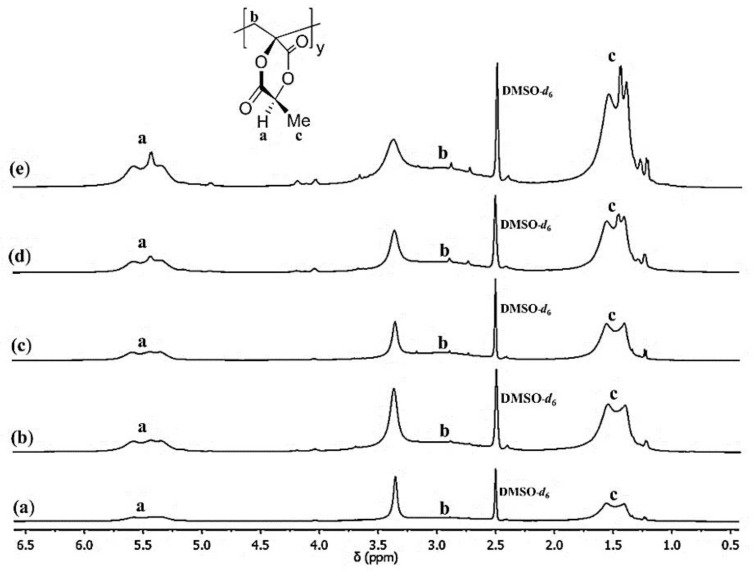
^1^H-NMR (400 MHz, DMSO-*d*_6_) spectra of poly(methylenelactide) with different amounts of AIBN (5, 10, 15, 20, 25 mol%): (**a**) PMLA_5_; (**b**) PMLA_10_; (**c**) PMLA_15_; (**d**) PMLA_20_; and (**e**) PMLA_25_.

**Figure 3:**
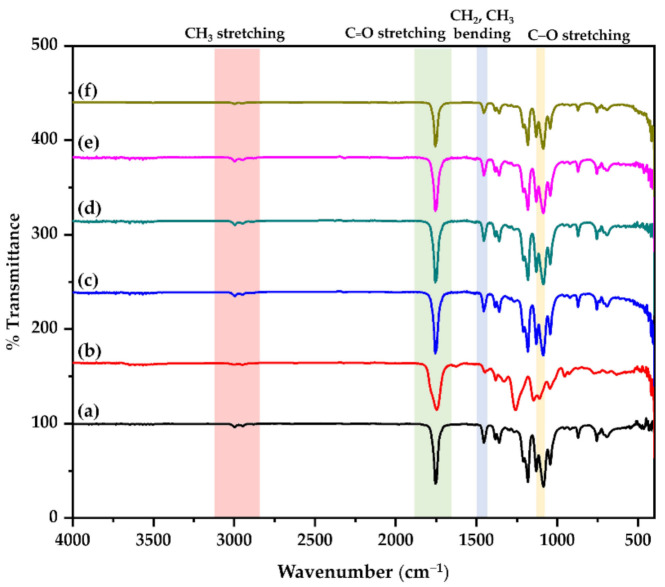
FT-IR spectra of homopolymers and graft copolymers with composition ratios of PMLA:LLA = 1:30, 1:50, 1:300, and 1:600 *w*/*w*: (**a**) PLA; (**b**) PMLA; (**c**) P(MLA20-*g*-LLA)_1:30_; (**d**) P(MLA20-*g*-LLA)_1:50_; (**e**) P(MLA20-*g*-LLA)_1:300_; and (**f**) P(MLA20-*g*-LLA)_1:600_.

**Figure 4:**
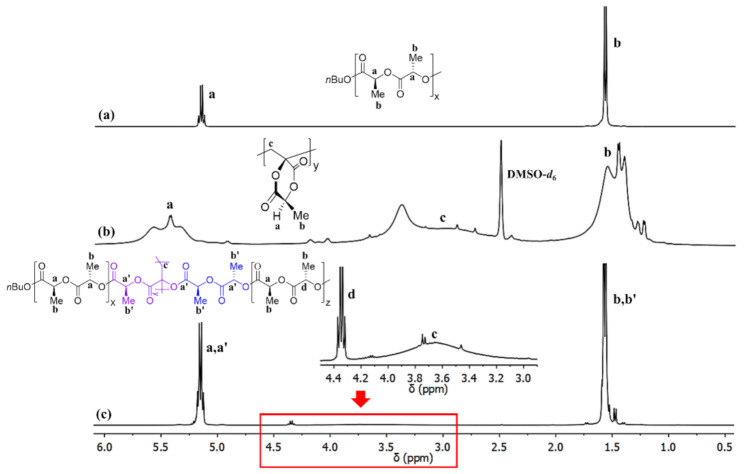
^1^H-NMR spectra of homopolymers and graft copolymers: (**a**) PLA (400 MHz, CHCl_3_-*d*_1_); (**b**) PMLA (400 MHz, DMSO-*d*_6_); and (**c**) P(MLA-*g*-LLA) (400 MHz, CHCl_3_-*d*_1_).

**Figure 5:**
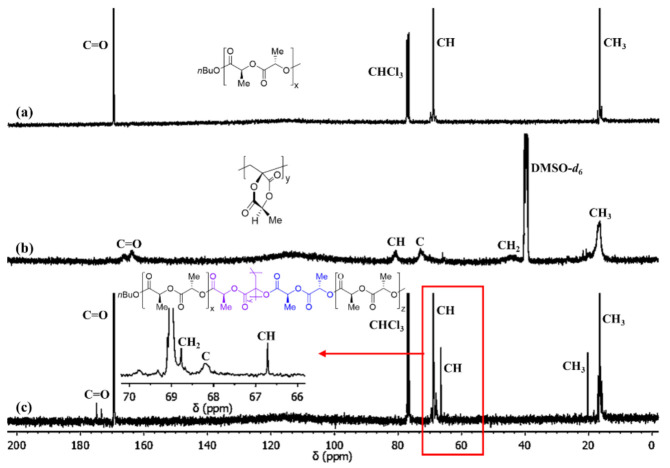
^13^C-NMR spectra of homopolymers and graft copolymers: (**a**) PLA (100 MHz, CHCl_3_-*d*_1_); (**b**) PMLA (100 MHz, DMSO-*d*_6_); and (**c**) P(MLA-*g*-LLA) (100 MHz, CHCl_3_-*d*_1_).

The chemical structure of PMLA, PLA, and P(MLA-*g*-LLA) were characterized by FT-IR and NMR. The FT-IR and NMR (^1^H, ^13^C) spectra of homopolymers and graft copolymers are as shown in Figure 1, Figure 2, Figure 3, Figure 4 and Figure 5. Some selected data for characterization of PMLA, PLA and P(MLA-*g*-LLA) are shown in Table 1.

FT-IR spectra of PMLA with different amounts of AIBN (5, 10, 15, 20, and 25 mol%) are shown in Figure 1. The spectrum of PMLA shows vibrational peaks of C=O and C–O stretching at around 1747 and 1110 cm^−1^, while the stretching and bending vibrations of C–H are seen at around 2949–2950 and 1447 cm^−1^. The PLA shows vibrational bands due to C–O stretching around 1085 cm^−1^, C=O stretching at 1754 cm^−1^ and C–H bending and stretching at 1455 and 2997 cm^−1^, respectively. Figure 3 shows the FT-IR spectra of PMLA, PLA and P(MLA20-*g*-LLA) with various PMLA:LLA composition ratios respectively. In comparison, the P(MLA-*g*-LLA) spectrum shows the characteristic peaks of C–O stretching around 1087 cm^−1^, C=O stretching around 1754 cm^−1^ and C–H bending and stretching at 1455 and 2997 cm^−1^, similar to the data reported for PLA [4].

The MLA conversion with different amounts of AIBN (5, 10, 15, 20, 25 mol%) was determined via ^1^H-NMR spectroscopy (Figure 2). The intensity of the NMR peak of PMLA increased according to the amount of AIBN. The comparison ^1^H-NMR spectra of PLA, PMLA and P(MLA-*g*-LLA) are shown in Figure 4. As expected, the methane proton (-CH) at 5.45–5.46 ppm of PMLA and 5.15–5.16 ppm of P(MLA-*g*-LLA) and PLA, methyl proton (-CH_3_) at 1.41–1.43 ppm of PMLA, and 1.57–1.58 ppm of P(MLA-*g*-LLA) and PLA structure were observed accordingly. In Figure 4, ^1^H-NMR peak of P(MLA-*g*-LL) around 3.0–4.3 ppm are very similar to the methyl proton of PMLA. From ^1^H-NMR spectrum combined with ^13^C-NMR spectrum (Figure 4 and Figure 5), the results confirmed the grafted structure of P(MLA-*g*-LLA). The black and blue units show PLA part on P(MLA-*g*-LLA) structure whereas the violet unit shows PMLA part on the backbone of the graft copolymer structure. ^13^C-NMR spectroscopy was further employed to provide a better view on the electron density of quaternary carbon and methylene carbon atom. From the results obtained (Figure 5), the quaternary carbon atoms of PMLA at 80.76 ppm and P(MLA-*g*-LLA) at 68.18 ppm were observed. This methylene carbon atom shows a relatively upfield in the case of PMLA at 45.08 ppm compared to the downfield in P(MLA-*g*-LLA) at 68.77 ppm. The electron-withdrawing of the ester group and oxygen atom influences preferentially the methylene carbon atom of P(MLA-*g*-LLA).

**Figure 6:**
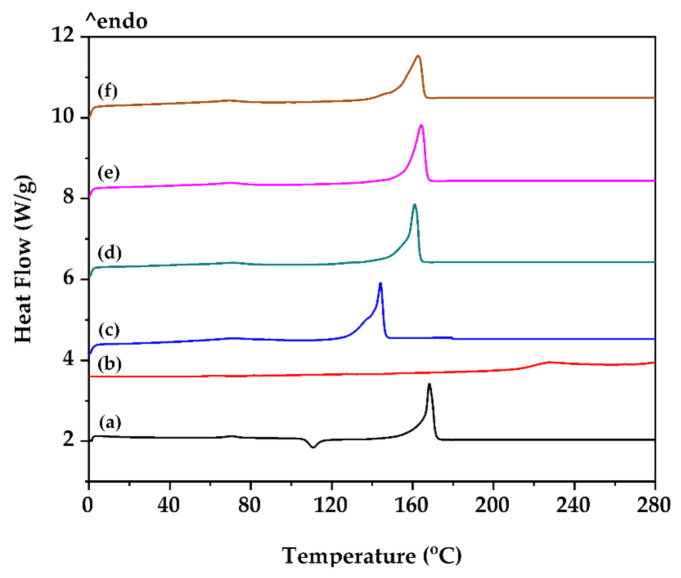
DSC thermograms (2nd scan) of PLA and P(MLA20-*g*-LLA) with composition ratio of PMLA:LLA of: 1:30, 1:50, 1:300, and 1:600 *w*/*w*: (**a**) PLA; (**b**) PMLA_20_; (**c**) P(MLA20-*g*-LLA)_1:30_; (**d**) P(MLA20-*g*-LLA)_1:50_; (**e**) P(MLA20-*g*-LLA)_1:300_; and (**f**) P(MLA20-*g*-LLA)_1:600_.

**Figure 7:**
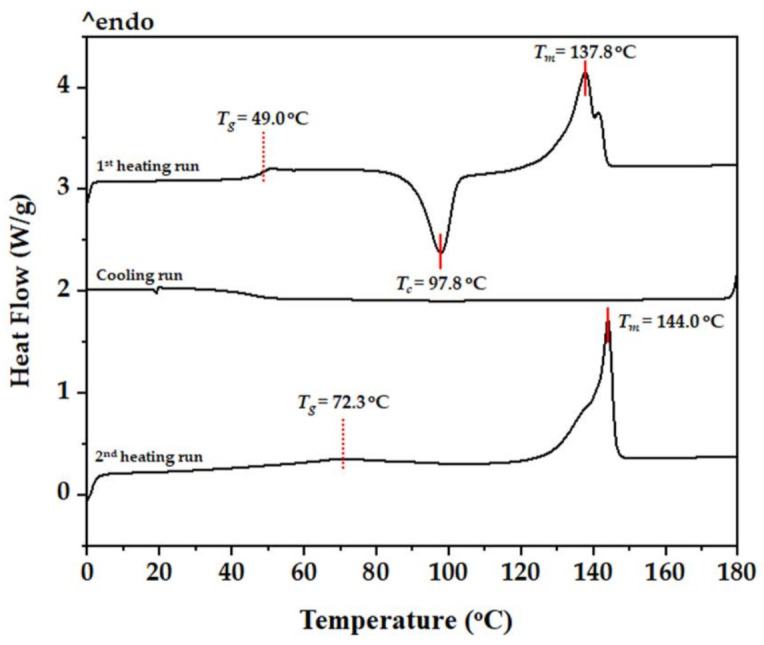
Representative DSC heating/cooling/heating thermograms of the P(MLA20-*g*-LLA)_1:30_ showing its *T*_g_, *T*_c_, and *T*_m_.

The DSC and TGA data of PMLA, PLA and P(MLA-*g*-LLA) are summarized in Table 1. From Figure 6, DSC thermograms of all PMLA obtained from using AIBN at different concentrations as an initiator show rather high glass transition temperature (*T*_g_) at about 221 to 225 °C. *T*_g_ of PMLA decreased as the amount of AIBN in PMLA increased from 5 to 25 mol%. No melting temperature (*T*_m_) was observed due to the amorphous structure of the PMLA which is in good agreement with the previously published work [48]. However, when it comes to the P(MLA-*g*-LLA) graft copolymers, the melting temperature of about 144–164 °C was detected. Figure 7 shows the heating-cooling-heating cycles of the DSC measurement of the P(MLA20-*g*-LLA) with a ratio of 1:30 *w*/*w*. For this P(MLA-*g*-LLA)_1:30_, in comparison to other P(MLA-*g*-LLA)s with different composition ratios, the lowest melting temperature of approximately 144 °C was observed. Also, it can be seen that for all cases, excluding P(MLA-*g*-LLA)_1:600_, *T*_m_ of the graft copolymer increased as the amount of LLA increased (Figure 6 and Table 1).

**Figure 8:**
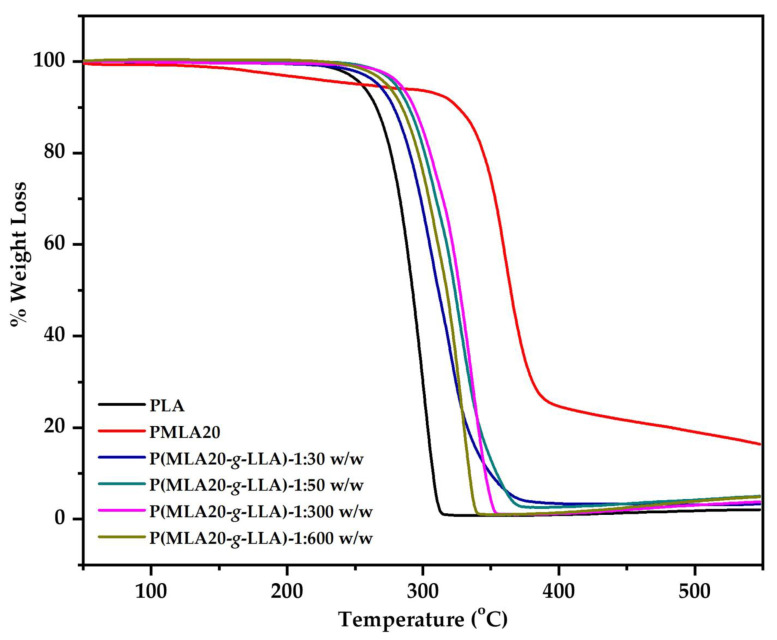
TG thermograms of PLA, PMLA_20_, and P(MLA20-*g*-LLA) with composition ratio of PMLA:LLA = 1:30, 1:50, 1:300, and 1:600 *w*/*w*.

As shown in Figure 8, the TGA thermogram of the PLA sample displays a decomposition onset temperature (*T*_onset_) of 210 °C and an end temperature (*T*_end_) of 315 °C with about 98% weight loss. In comparison, the P(MLA-*g*-LLA) graft copolymers with composition ratios of PMLA:LLA of 1:30, 1:50, 1:300, and 1:600 *w*/*w* show onset temperatures of around 200–245 °C with degradation complete at ~344–400 °C and ~96–98%weight loss. The results show that the presence of PMLA in the P(MLA-*g*-LLA) structure increases the decomposition temperature, therefore, an improvement of thermal stability, comparing with the *T*_d_ value of PLA alone.

**Figure 9:**
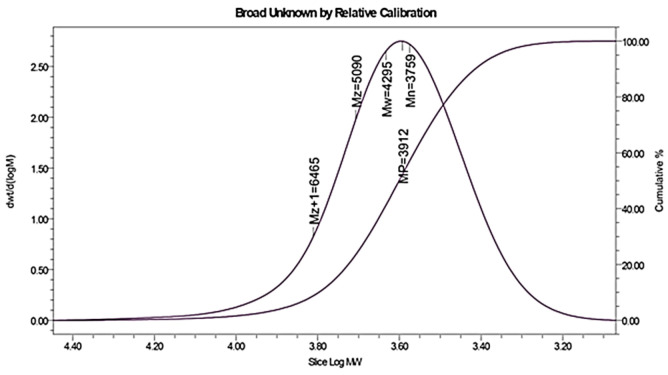
GPC chromatogram of the P(MLA20-*g*-LLA)_1:30_ showing the average molecular weight values of: ${\bar{M}}_{Z+1}$, ${\bar{M}}_{Z}$, ${\bar{M}}_{w}$, and ${\bar{M}}_{n}$, respectively.

For the molar mass characterization, the number- and weight-average molecular weights (${\bar{M}}_{n}\;\mathrm{and}\;{\bar{M}}_{w}$) and dispersity (*Đ* = ${\bar{M}}_{w}/{\bar{M}}_{n}$) of the polymers were determined by gel permeation chromatography (GPC) using THF as a solvent at 40 °C (flow rate = 1 mL min^−1^) and calibrated with polystyrene (PS) standards using Empower Software. From the results obtained (see Table 1), the graft copolymers showed rather low molecular weights with ${\bar{M}}_{w}$ ~ 4000–10,000 g mol^−1^ and narrow dispersity (*Đ* ~ 1.1–1.5). Figure 9 shows the GPC chromatogram of P(MLA20-*g*-LLA)_1:30_ graft copolymer which the ${\bar{M}}_{w},\;{\bar{M}}_{n},\;\mathrm{and}$ *Đ* values of 4.30 × 10^3^, 3.76 × 10^3^, and 1.1 were observed respectively.

### 3.7. PMLA via Vinyl-Addition Polymerization

The homopolymerization reactions of PMLA were carried out in the presence of 5, 10, 15, 20 and 25 mol% of AIBN at 75 °C in 1,2-dichlorobenzene (*o*-DCB). PMLA was synthesized via vinyl addition polymerization using AIBN as an initiator using the free radical to yield predominantly isotactic polymer structures [59] and PMLA product was obtained as a white solid in a percentage yield of 60–65%. From the results obtained, it was found that as the amount of AIBN increased, the molecular weight of the polymer decreased.

### 3.8. P(MLA-g-LLA) and PLA via Ring-Opening Polymerization

**Scheme 4:**
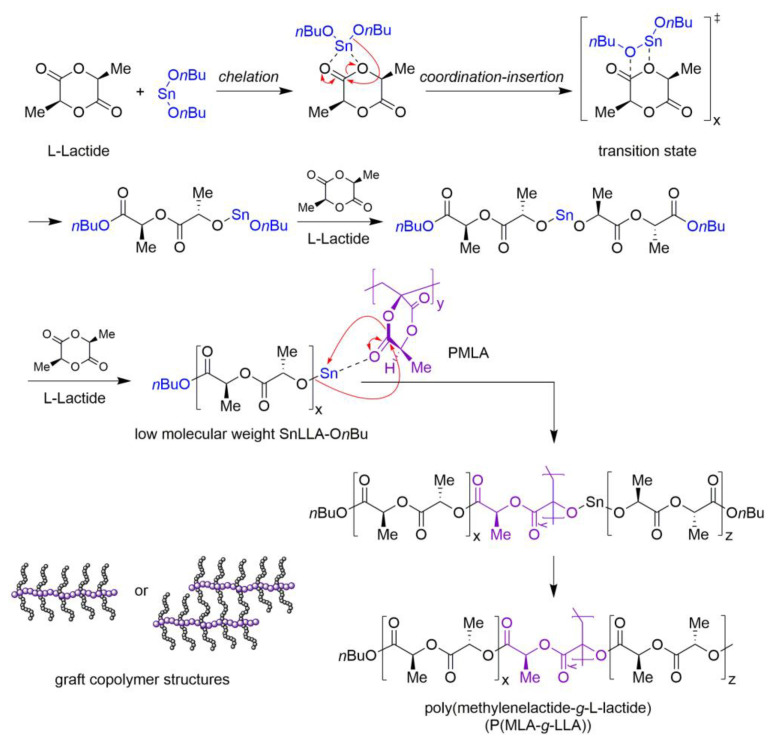
Proposed mechanism for the synthesis of poly(methylenelactide-*g*-l-lactide), P(MLA-*g*-LLA).

P(MLA-*g*-LLA) and PLA were synthesized via ring-opening polymerization (ROP). The optimal condition for the synthesis of P(MLA-*g*-LLA) and PLA was 0.03 mol% of liquid tin(II) *n*-butoxide at 120 °C for 72 h. The physical appearance of the purified P(MLA-*g*-LLA) and PLA were obtained as a white solid with percentage yield of synthesis around 70–90% and 90% respectively. P(MLA-*g*-LLA) was found to have a relatively low molecular weight of ${\bar{M}}_{w}$ ~ 4000–11,000 g mol^−1^ and a narrow dispersity (*Đ*) of ~1.1–1.4 compared to PLA as the data shown in Table 1. For P(MLA-*g*-LLA), it was synthesized via ROP by LLA initiated of ester group on PMLA chain with the proposed mechanisms as shown in Scheme 4.

From Scheme 4, the proposed mechanism of the synthesized poly(methylenelactide-*g*-L-lactide), P(MLA-*g*-LLA) starts with the O atoms of LLA have been activated by chelation with the Sn atom of Sn(O*n*Bu)_2_ initiator. Then, the reactive butoxy group of initiator attacks the carbonyl group of LLA through coordination-insertion to give *n*BuOSn–butyl lactate. Next, this intermediate was reacted with LLA in the propagation step to obtain the propagating species as low molecular weight SnLLA–O*n*Bu. After that, the Sn atom of low molecular weight SnLLA–O*n*Bu attacks the carbonyl carbon of PMLA chain and inserts into LLA ring of PMLA to form the P(MLA-*g*-LLA).

## 4. Conclusions

In this research work, the synthesis of P(MLA-*g*-LLA) graft copolymers were successfully performed by a combination of vinyl-addition and ring-opening polymerizations (ROP). The optimum condition for the synthesis of the graft copolymer was obtained from using 0.03 mol% of liquid Sn(O*n*Bu)_2_ at 120 °C for 72 h. The physical appearance of P(MLA-*g*-LLA) was obtained as a white solid with a percentage yield of approximately 90%. To investigate the polymerization behavior of P(MLA-*g*-LLA), different molar amounts of PMLA were used. The grafted structure of P(MLA-*g*-LLA) can be confirmed by a combination of ^1^H, ^13^C-NMR, and FT-IR techniques.

Thermal properties were characterized by DSC and TGA. The melting temperature (*T*_m_) of P(MLA-*g*-LLA) with PMLA:LLA ratios of 1:30 and 1:50 *w*/*w* in the range of about 144–164 °C was observed. From the DSC results, it can be seen that the *T*_m_ values of the copolymers decreased according to the amount of AIBN in PMLA from 5 to 25 mol%. The thermal decomposition temperature (*T*_d_) of around 328–386 °C from TGA was higher than that of the PLA homopolymer (~300 °C) indicating that the grafting reaction has increased their thermal stability due to the PMLA content of the graft copolymers.

These graft copolymers were obtained with the weight-average molecular weights (${\bar{M}}_{w})$ ~ 4000–11,000 g mol^−1^ and narrow dispersities (*Đ* = 1.1–1.4). It is considered that they could be incorporated as additives in commercial PLA to improve both its melt processing characteristics and various thermo-mechanical properties. Such improvements would further broaden the range of applications of PLA as a bioplastic.

## Figures and Tables

**Table 1:** Selected characterization data of PMLA, PLA, and P(MLA-*g*-LLA).

Sample	Initiator	Composition Ratio PMLA:LLA (*w*/*w*)	*T*_g_ (°C) *^a^*	*T*_c_ (°C) *^a^*	*T*_m_ (°C) *^a^*	*T*_d_ (°C) *^b^*	${\bar{M}}_{w}$ (g/mol) *^c^*	${\bar{M}}_{n}$ (g/mol) *^c^*	Dispersity (*Đ*) $({\bar{M}}_{w}/{\bar{M}}_{n})$ *^c^*
PMLA-5	AIBN	-	244.0	-	-	368.0	-	-	-
PMLA-10	AIBN	-	225.7	-	-	360.1	-	-	-
PMLA-15	AIBN	-	223.7	-	-	361.1	-	-	-
PMLA-20	AIBN	-	221.4	-	-	356.9	-	-	-
PMLA-25	AIBN	-	220.3	-	-	356.4	-	-	-
PLA	Sn(O*n*Bu)_2_	-	71.5	110.8	168.3	301.1	1.68 × 10^4^	1.11 × 10^4^	1.5
P(MLA5-*g*-LLA)	Sn(O*n*Bu)_2_	1:30	70.1	-	159.8	318.5	9.04 × 10^4^	6.78 × 10^3^	1.3
P(MLA5-*g*-LLA)	Sn(O*n*Bu)_2_	1:50	73.3	-	164.2	328.8	1.14 × 10^4^	8.24 × 10^3^	1.4
P(MLA10-*g*-LLA)	Sn(O*n*Bu)_2_	1:30	70.6	-	155.5	318.5	7.08 × 10^3^	5.66 × 10^3^	1.2
P(MLA10-*g*-LLA)	Sn(O*n*Bu)_2_	1:50	74.8	-	159.7	325.3	8.74 × 10^3^	6.63 × 10^3^	1.3
P(MLA15-*g*-LLA)	Sn(O*n*Bu)_2_	1:30	73.4	-	155.8	321.4	6.11 × 10^3^	5.15 × 10^3^	1.2
P(MLA15-*g*-LLA)	Sn(O*n*Bu)_2_	1:50	66.8	-	160.3	326.8	8.21 × 10^3^	6.34 × 10^3^	1.3
P(MLA20-*g*-LLA)	Sn(O*n*Bu)_2_	1:30	72.3	-	144.0	314.4	4.30 × 10^3^	3.76 × 10^3^	1.1
P(MLA20-*g*-LLA)	Sn(O*n*Bu)_2_	1:50	73.0	-	161.0	324.1	8.45 × 10^3^	6.48 × 10^3^	1.3
P(MLA25-*g*-LLA)	Sn(O*n*Bu)_2_	1:30	73.6	-	163.3	328.1	4.87 × 10^3^	4.08 × 10^3^	1.2
P(MLA25-*g*-LLA)	Sn(O*n*Bu)_2_	1:50	73.5	-	163.8	386.7	4.59 × 10^3^	3.81 × 10^3^	1.2
P(MLA20-*g*-LLA)	Sn(O*n*Bu)_2_	1:300	68.8	-	164.3	335.4	1.01 × 10^4^	7.86 × 10^3^	1.4
P(MLA20-*g*-LLA)	Sn(O*n*Bu)_2_	1:600	68.4	-	162.8	328.9	1.10 × 10^4^	7.88 × 10^3^	1.3

*^a^* Determined by differential scanning calorimetry (DSC), *T*_g_; *T*_c_; and *T*_m_ values obtained from the 2nd scan; *^b^* Determined by thermal gravimetric analysis (TGA), *T*_d_ observed from peak maximum of the thermal decomposition, temperature; and *^c^* Weight-average molecular weight (${\bar{M}}_{w}$), number-average molecular weight (${\bar{M}}_{n}$), and dispersity (*Đ*) determined by gel permeation chromatography (GPC) using a 2414 RI detector and PS standard (Empower 3 Software).

## References

[1] Castillo Martinez F.A., Balciunas E.M., Salgado J.M., Domínguez González J.M., Converti A., Oliveira R.P.d.S. (2013). Lactic acid Properties, Applications and Production: A review. Trends Food Sci. Technol..

[2] Carothers W.H., Dorough G.L., van Natta F.J. (1932). Studies of Polymerization and Ring Formation. X. The Reversible Polymerization of Six-Membered Cyclic Esters. J. Am. Chem. Soc..

[3] Hartmann M.H. (1998). High Molecular Weight Polylactic Acid Polymers. Biopolymers from Renewable Resources.

[4] Garlotta D. (2001). A Literature Review of Poly(Lactic Acid). J. Polym. Environ..

[5] Kricheldorf H.R. (2001). Syntheses and Application of Polylactides. Chemosphere.

[6] Gupta A.P., Kumar V. (2007). New Emerging Trends in Synthetic Biodegradable Polymers-Polylactide: A Critique. Eur. Polym. J..

[7] Groot W., van Krieken J., Sliekersl O., de Vos S. (2010). Production and Purification of Lactic acid and Lactide. Poly(lactic acid) Synthesis, Structures, Properties, Processing, and Applications.

[8] Dechy-Cabaret O., Martin-Vaca B., Bourissou D. (2009). Polyesters from Dilactones. Handbook of Ring-Opening Polymerization.

[9] Castro-Aguirre E., Iñiguez-Franco F., Samsudin H., Fang X., Auras R. (2016). Poly(lactic acid)-Mass Production, Processing, Industrial Applications, and End of Life. Adv. Drug. Deliv. Rev..

[10] Lee C., Hong S. (2014). An Overview of the Synthesis and Synthetic Mechanism of Poly (Lactic acid). Mod. Chem. Appl..

[11] Nofar M., Sacligil D., Carreau P.J., Kamal M.R., Heuzey M.-C. (2019). Poly (lactic acid) blends: Processing, Properties and Applications. Int. J. Biol. Macromol..

[12] Zhou Y., Huang Z., Diao X., Weng Y., Wang Y. (2015). Characterization of the Effect of REC on the Compatibility of PHBH and PLA. Polym. Test..

[13] Hsu Y.I., Masutani K., Yamaoka T., Kimura Y. (2015). Strengthening of Hydrogels made from Enantiomeric Block Copolymers of Polylactide (PLA) and Poly(ethylene glycol) (PEG) by the Chain Extending Diels-Alder Reaction at the Hydrophilic PEG Terminals. Polymer.

[14] Al-Itry R., Lamnawar K., Maazouz A., Billon N., Combeaud C. (2015). Effect of the Simultaneous Biaxial Stretching on the Structural and Mechanical Properties of PLA, PBAT and their Blends at Rubbery State. Eur. Polym. J..

[15] Zuo H., Liu J., Huang D., Bai Y., Cui L., Pan L., Zhang K., Wang H. (2021). Sustainable and High-Performance Ternary Blends from Polylactide, CO_2_-Based Polyester and Microbial Polyesterswith Different Chemical Structure. J. Polym. Sci..

[16] Chen J., Zhang T.-Y., Jin F.-L., Park S.-J. (2018). Fracture Toughness Improvement of Poly(lactic acid) Reinforced with Poly(*ε*-caprolactone) and Surface-Modified Silicon Carbide. Adv. Mater. Sci. Eng..

[17] Liu X., Yu L., Dean K., Toikka G., Bateman S., Nguyen T., Yuan Q., Filippou C. (2013). Improving Melt Strength of Polylactic Acid. Int. Polym. Process..

[18] Quiles-Carrillo L., Duart S., Montanes N., Torres-Giner S., Balart R. (2018). Enhancement of the Mechanical and Thermal Properties of Injection-Molded Polylactide Parts by the Addition of Acrylated Epoxidized Soybean Oil. Mater. Des..

[19] Herrera N., Roch H., Salaberria A.M., Pino-Orellana M.A., Labidi J., Fernandes S.C.M., Radic D., Leiva A., Oksman K. (2016). Functionalized Blown Films of Plasticized Polylactic Acid/Chitin Nanocomposite: Preparation and Characterization. Mater. Des..

[20] Wojtczak E., Kubisa P., Bednarek M. (2018). Thermal Stability of Polylactide with Different End-Groups Depending on the Catalyst used for the Polymerization. Polym. Degrad. Stab..

[21] Nagarajan V., Mohanty A.K., Misra M. (2016). Perspective on Polylactic Acid (PLA) Based Sustainable Materials for Durable Applications: Focus on Toughness and Heat Resistance. ACS Sustain. Chem. Eng..

[22] Jin F.-L., Hu R.-R., Park S.-J. (2018). Improvement of Thermal Behaviors of Biodegradable Poly(lactic acid) Polymer: A Review. Composites Part B: Engineering. Compos. B Eng..

[23] Wu F., Misra M., Mohanty A.K. (2019). Studies on Why the Heat Deflection Temperature of Polylactide Bioplastic cannot be Improved by Overcrosslinking. Polym. Cryst..

[24] Södergård A., Stolt M. (2002). Properties of Lactic Acid Based Polymers and their Correlation with Composition. Prog. Polym. Sci..

[25] Zhang Z.C., Gao X.R., Hu Z.J., Yan Z., Xu J.Z., Xu L., Zhong G.J., Li Z.M. (2016). Inducing Stereocomplex Crystals by Template Effect of Residual Stereocomplex Crystals during Thermal Annealing of Injection-Molded Polylactide. Ind. Eng. Chem. Res..

[26] Deng L., Xu C., Wang X., Wang Z. (2017). Supertoughened Polylactide Binary Blend with High Heat Deflection Temperature Achieved by Thermal Annealing above the Glass Transition Temperature. ACS Sustain. Chem. Eng..

[27] Al-Itry R., Lamnawar K., Maazouz A. (2012). Improvement of Thermal Stability, Rheological and Mechanical Properties of PLA, PBAT and their Blends by Reactive Extrusion with Functionalized Epoxy. Polym. Degrad. Stab..

[28] Barczewski M., Mysiukiewicz O., Matykiewicz D., Kloziński A., Andrzejewski J., Piasecki A. (2020). Synergistic Effect of Different Basalt Fillers and Annealing on the Structure and Properties of Polylactide Composites. Polym. Test..

[29] Purnama P., Samsuri M., Iswaldi I. (2021). Properties Enhancement of High Molecular Weight Polylactide Using Stereocomplex Polylactide as a Nucleating Agent. Polymers.

[30] Zhang B., Sun B., Bian X., Li G., Chen X. (2016). High Melt Strength and High Toughness PLLA/PBS Blends by Copolymerization and In Situ Reactive Compatibilization. Ind. Eng. Chem. Res..

[31] Safandowska M., Rozanski A., Galeski A. (2020). Plasticization of Polylactide after Solidification:An Effectiveness and Utilization for Correct Interpretation of Thermal Properties. Polymers.

[32] Purnama P., Kim S.H. (2014). Bio-Based Composite of Stereocomplex Polylactide and Cellulose Nanowhiskers. Polym. Degrad. Stab..

[33] Yang S., Wu Z.-H., Yang W., Yang M.-B. (2008). Thermal and Mechanical Properties of Chemical Crosslinked Polylactide (PLA). Polym. Test..

[34] Lou X., Detrembleur C., Jérôme R. (2003). Novel Aliphatic Polyesters Based on Functional Cyclic(Di)Esters. Macromol. Rapid Commun..

[35] Cheng Y., Deng S., Chen P., Ruan R. (2009). Polylactic Acid (PLA) Synthesis and Modifications: A Review. Front. Chem. China.

[36] Agarwal S., Jin Q., Maji A. (2012). Biobased Polymers from Plant-Derived Tulipalin A. J. Am. Chem. Soc..

[37] Castillejos S., Cerna J., Meléndez F., Castro M.E., Aguilar R., Beltrán C.M., González M. (2018). Bulk Modification of Poly(lactide) (PLA) via Copolymerization with Poly(propylene glycol) Diglycidylether (PPGDGE). Polymers.

[38] Dechy-Cabaret O., Martin-Vaca B., Bourissou D. (2004). Controlled Ring-Opening Polymerization of Lactide and Glycolide. Chem. Rev..

[39] Nuyken O., Pask S.D. (2013). Ring-Opening Polymerization-An Introductory Review. Polymers.

[40] Hagiopol C. (1999). Binary Copolymerization. Copolymerization: Toward a Systematic Approach.

[41] Osswald T.A., Menges G. (2012). Structure of Polymers. Materials Science of Polymers for Engineers.

[42] Kaliva M., Georgopoulou A., Dragatogiannis D.A., Charitidis C.A., Chatzinikolaidou M., Vamvakaki M. (2020). Biodegradable Chitosan-graft-Poly(l-lactide) Copolymers For Bone Tissue Engineering. Polymers.

[43] Xia L., Zhang Z., Hong C.Y., You Y.Z. (2019). Synthesis of Copolymer via Hybrid Polymerization: From Random to Well-Defined Sequence. Eur. Polym. J..

[44] Stannett V. (1981). Grafting. Radiat. Phys. Chem..

[45] Athawale V.D., Rathi S.C. (1999). Graft Polymerization: Starch as a Model Substrate. J. Macromol. Sci. Part C.

[46] Deng Y., Zhang S., Lu G., Huang X. (2013). Constructing Well-Defined Star Graft Copolymers. Polym. Chem..

[47] Gowda R.R., Chen E.Y.-X. (2013). Sustainable Polymers from Biomass-Derived α-Methylene-γ-Butyrolactones. Encyclopedia of Polymer Science and Technology.

[48] Miyake G.M., Zhang Y., Chen E.Y.-X. (2015). Polymerizability of *Exo*-Methylene-Lactide Toward Vinyl Addition and Ring Opening. J. Polym. Sci. A Polym. Chem..

[49] Stridberg K.M., Ryner M., Albertsson A.C. (2002). Controlled Ring-Opening Polymerization: Polymers with Designed Macromolecular Architecture. Adv. Polym. Sci..

[50] Yin M., Baker G.L. (1999). Preparation and Characterization of Substituted Polylactides. Macromolecules.

[51] Trimaille T., Möller M., Gurny R. (2004). Synthesis and Ring-Opening Polymerization of New Monoalkyl-Substituted Lactides. J. Polym. Sci. A Polym. Chem..

[52] Marcincinova Benabdillah K., Boustta M., Coudane J., Vert M. (2001). Novel Degradable Polymers Combining D-Gluconic Acid, a Sugar of Vegetal Origin, with Lactic and Glycolic Acids. Biomacromolecules.

[53] Such G.K., Quinn J.F., Quinn A., Tjipto E., Caruso F. (2006). Assembly of Ultrathin Polymer Multilayer Films by Click Chemistry. J. Am. Chem. Soc..

[54] Jing F., Hillmyer M.A. (2008). A Bifunctional Monomer Derived from Lactide for Toughening Polylactide. J. Am. Chem. Soc..

[55] Fiore G.L., Jing F., Young V.G., Cramer C.J., Hillmyer M.A. (2010). High *T*_g_ Aliphatic Polyesters by the Polymerization of Spirolactide Derivatives. Polym. Chem..

[56] Castillo J.A., Borchmann D.E., Cheng A.Y., Wang Y., Hu C., García A.J., Weck M. (2012). Well-Defined Poly(lactic acid)s Containing Poly(ethylene glycol) Side Chains. Macromolecules.

[57] Beuille E., Darcos V., Coudane J., Lacroix-Desmazes P., Nottelet B. (2013). Regioselective Halogenation of Poly(lactide) by Free-Radical Process. Macromol. React. Eng..

[58] Britner J., Ritter H. (2015). Self-Activation of Poly(methylenelactide) through Neighboring-Group Effects: A Sophisticated Type of Reactive Polymer. Macromolecules.

[59] Britner J., Ritter H. (2016). Methylenelactide: Vinyl polymerization and spatial reactivity effects. Beilstein J. Org. Chem..

[60] Zhu J.B., Tang X., Falivene L., Caporaso L., Cavallo L., Chen E.Y.-X. (2017). Organocatalytic Coupling of Bromo-Lactide with Cyclic Ethers and Carbonates to Chiral Bromo-Diesters: NHC or Anion Catalysis?. ACS Catal..

[61] Mauldin T.C., Wertz J.T., Boday D.J. (2016). Acrylic Platform from Renewable Resources via a Paradigm Shift in Lactide Polymerization. ACS Macro Lett..

[62] Sinclair F., Shaver M.P. (2018). Cross-Metathesis Functionalized Exo-Olefin Derivatives of Lactide. J. Polym. Sci. A Polym. Chem..

[63] Boday D.J., Garcia J.M., Hedrick J.L., Kobilka B.M., Wertz J.T., Wojtecki R.J. (2019). Lactide-Derived Polymers with Improved Materials Properties via Polyhexahydrotriazines (PHT) Reaction. U.S. Patent.

[64] Meepowpan P., Punyodom W., Molloy R. (2017). Process for the Preparation of Liquid Tin(II) Alkoxides. U.S. Patent.

[65] Chaiwon T., Sriyai M., Meepowpan P., Molloy R., Nalampang K., Kaabbuathong N., Punyodom W. (2021). Kinetic and Mechanistic Studies of Bulk Copolymerization of L-lactide and Glycolide Initiated by Liquid Tin(II) *n*-Butoxide. Chiang Mai J. Sci..

[66] Sriyai M., Chaiwon T., Molloy R., Meepowpan P., Punyodom W. (2020). Efficiency of Liquid Tin(II) *n*-Alkoxide Initiators in the Ring-Opening Polymerization of L-lactide: Kinetic Studies by Non-Isothermal Differential Scanning Calorimetry. RSC Adv..

[67] Sattayanon C., Sontising W., Jitonnom J., Meepowpan P., Punyodom W., Kungwan N. (2014). Theoretical Study on the Mechanism and Kinetics of Ring-Opening Polymerization of Cyclic Esters Initiated by Tin(II) *n*-Butoxide. Comput. Theor. Chem..

[68] Dumklang M., Pattawong N., Punyodom W., Meepowpan P., Molloy R., Hoffman M. (2009). Novel Tin(II) Butoxides for Use as Initiators in the Ring-Opening Polymerisation of *ε*-Caprolactone. Chiang Mai J. Sci..

